# Evaluating the Effects of Replacing Alfalfa with *Broussonetia papyrifera* Branch/Leaf Powder on Growth and Serum Indicators in Dezhou Donkeys

**DOI:** 10.3390/ani14010123

**Published:** 2023-12-29

**Authors:** Yongguang Chen, Boying Dong, Honglei Qu, Jie Cheng, Yulong Feng, Lilin Liu, Qiugang Ma

**Affiliations:** 1 State Key Laboratory of Animal Nutrition, College of Animal Science and Technology, China Agricultural University, Beijing 100193, China; 2 Key Laboratory of Tarim Animal Husbandry Science and Technology, College of Animal Science, Tarim University, Alar 843300, China; 3 National Engineering Research Center for Gelatin-Based Traditional Chinese Medicine, Dong-E-E-Jiao Co., Ltd., Liaocheng 252200, China

**Keywords:** *Broussonetia papyrifera* branch/leaf powder, nutrients digestibility, growth performance, serum indicators, donkey

## Abstract

**Simple Summary:**

In recent years, with the expansion of animal husbandry, the competition for grain between its use as feedstuffs for animals and as foodstuffs for humans has become increasingly prominent. Traditional feeds such as corn, soybean, and alfalfa are no longer sufficient to meet the growing needs of the livestock industry. Consequently, it is imperative to develop novel, unconventional feedstuffs. *Broussonetia papyrifera* (BP) emerges as a promising substitute, being a woody feedstuff with wide environmental adaptability, high yield, and high protein content.

**Abstract:**

The purpose of this experiment was to study the apparent digestibility and the effects of *Broussonetia papyrifera* (BP) branch/leaf powder supplementation on growth performance and serum indicators in donkeys. The results showed that the apparent digestibility of dry matter (DM), crude protein (CP), crude fiber (CF), neutral detergent fiber (NDF), acidic detergent fiber (ADF), and digestible energy content (DE) of BP branch/leaf powder were 51.88%, 67.27%, 64.86%, 49.59%, 54.73%, 40.87%, and 6.37 MJ/kg, respectively. The average daily gain (ADG) in the 20% group was significantly higher than in the 0% and 30% groups. The serum albumin (ALB) levels in the 0% and 10% groups were significantly higher than those in the 20% and 30% groups, while the serum globulin (GLB) content in the 10% group was significantly lower than in the other groups. The 20% group showed decreased serum triglyceride (TG) levels compared to the other groups. Both the 20% and 30% groups exhibited lower total cholesterol (TC) levels and increased alanine aminotransferase (ALT) compared to the 0% and 10% groups and higher serum lactate dehydrogenase (LDH) levels than the 10% group. The 30% group had higher serum immunoglobulin A (IgA) levels than the other groups, while all three BP branch/leaf powder groups had lower serum tumor necrosis factor (TNF-α) levels than the 0% group. There was a gradual increase in serum total antioxidant capacity (T-AOC) with the increasing amount of BP branch/leaf powder added. In conclusion, the optimal supplemental proportion of BP branch/leaf powder in the diet is 20%. Furthermore, BP branch/leaf powder can improve growth performance, serum immune indices, and antioxidant capacity in Dezhou donkeys.

## 1. Introduction

The Dezhou donkey, a large breed of donkey in China, has excellent traits such as tall stature, tolerance to rough feeding, strong adaptability, and stable genetic performance. Donkeys are a special economic animal with high meat value, dairy value, and medicinal value. Donkey meat has a high protein content, rich in essential amino acids and unsaturated fatty acids, and low fat content, making it a healthy health food [1,2]. Donkey milk has a low fat content compared to cow milk, but it contains up to 30.7% of essential fatty acids (such as linoleic acid) [3,4]. Donkey milk contains bioactive chemicals, especially lipids, which directly or indirectly regulate the immune system in the intestinal environment, making it a healthy food [5,6]. Donkey skin is the main raw material for making *Asini Corii Colla*, which is a good tonic product and is popular with consumers. The donkey industry has great development prospects, and it is imperative to develop new types of feed materials to promote the sustainable development of the donkey industry.

Alfalfa, a leguminous plant high in crude protein and fiber, can be harvested multiple times [7,8]. It is known as the king of forage and is widely used in animal husbandry. However, due to differences in climate and soil in different regions, the nutritional composition of alfalfa is variable [9,10,11]. Recently, as animal husbandry expands, the competition between grains for feed and grains for human consumption has become increasingly prominent. Consequently, more fertile land is allocated for grain rather than alfalfa cultivation, leading to unstable alfalfa quality. This instability is often due to cultivation on unsuitable soil, and the price of high-quality alfalfa is rising. Therefore, developing a new type of high-protein forage to replace alfalfa is necessary.

*Broussonetia papyrifera* (BP), a member of the mulberry family, is a perennial deciduous woody species. It is primarily found in China, Vietnam, Japan, and Korea, with limited cultivation in Europe and the Americas [12]. As a multifunctional and comprehensive tree species, BP is widely used in the paper industry [13], pharmaceutical industry [14,15], and animal husbandry [16]. It is nutrient-rich, with its branch/leaf dry matter containing over 18% crude protein, phosphorus, and minerals, making it a viable protein feed [17]. It has the potential to alleviate the lack of protein feedstuff [13]. It is reported that BP improved the meat quality traits of fattening lamb, in which the diameter and area of muscle fibers were the smallest in the group 100%, and the beneficial fatty acid content was the highest and the harmful fatty acid content was the lowest in the group 18% [18]. In grass carp, adding 5% BP had no effect on growth, but 10% BP improved muscle quality by increasing muscle hardness and reducing fat accumulation and muscle fiber diameter, albeit with reduced growth performance [19]. BP silage supplementation in Holstein heifers affected serum antioxidant and immune indices: increasing BP silage led to lower serum malondialdehyde (MDA) and interleukin-1 β (IL-1β) levels and higher immunoglobulin A (IgA) and IL-4 levels [20]. Additionally, adding 15% BP silage improved performance in animals, increasing final body weight, average daily gain, dry matter intake, feed conversion rate, blood superoxide dismutase (SOD), and total antioxidant capacity (TAC), while reducing blood MDA and enhancing antioxidant capacity [21]. It is widely used in animal husbandry, but there are few studies of BP in donkeys.

Therefore, this experiment aims to explore the effects of gradient BP branch/leaf powder replacing alfalfa on the growth performance and serum biochemical, immune, and antioxidant indicators in Dezhou donkeys, providing a reference for BP’s application in donkey diets.

## 2. Materials and Methods

The animal study protocol was approved by the Ethics Committee of the College of Animal Science and Technology of China Agricultural University (Beijing, China) on 26 May 2023 under permit AW62503202-1-1.

### 2.1. Digestion Trial

Six genetically uniform male Dezhou donkeys, each with an initial body weight (IBW) of approximately 193 ± 10 kg and closely matched in terms of physique and health status, were selected for the study. Twenty mobile nylon bags were introduced into the stomachs of the donkeys via nasogastric intubation, assisted by a guide wire. Each mobile nylon bag, measuring 4 cm × 1 cm, was sealed using a sealing machine. After sealing three sides of the bag, it was turned inside out. Approximately 1 g of BP branch/leaf powder was weighed and placed into each clean mobile nylon bag, which was then numbered and sealed. These bags, containing the BP branch/leaf powder, were subsequently introduced into the donkeys’ stomachs. The procedure involved initially restraining the donkey, and then lubricating the inside and outside of the nasogastric tube with liquid paraffin. The tube was carefully inserted into the donkey’s esophagus through its nostrils. Finally, the mobile nylon bags were placed into the tube and pushed down into the stomach using the guide wire [22]. 

### 2.2. Feeding Trial

#### 2.2.1. Animals and Experimental Design

Thirty-two male Dezhou donkeys with an initial body weight (IBW) of 193 ± 10 kg were randomly divided into four groups, each consisting of eight replicates. They were fed a basal diet supplemented with 0%, 10%, 20%, and 30% *Broussonetia papyrifera* (BP) branch/leaf powder, forming the control group, 10% group, 20% group, and 30% group, respectively. The donkeys had access to their feed rations and water ad libitum throughout the experiment. All animals were treated with an anthelmintic two weeks prior to the start of the trial. The experiment lasted for 36 days.

#### 2.2.2. Animals Diet

The composition and nutrient levels of the four diets are presented in Table 1. The concentrate supplement was fed to each donkey twice daily, at 7:00 and 17:00, totaling 2.5 kg per day. Roughage was provided four times a day at 8:00, 12:00, 16:00, and 20:00. This included rice straw at a daily total of 1.0 kg, alfalfa hay in varying daily amounts of 1.5 kg, 1.0 kg, 0.5 kg, and 0 kg, and BP branch/leaf powder in corresponding daily amounts of 0 kg, 0.5 kg, 1.0 kg, and 1.5 kg, respectively. 

### 2.3. Sampling and Measurements

#### 2.3.1. Apparent Digestibility of BP Branch/Leaf Powder Measurement

The mobile nylon bags excreted by the donkey for the digestion trial were collected for three consecutive days. Then, the collected samples in the mobile nylon bags were processed. The collected mobile nylon bags were cleaned with water and dried to a constant weight at 65 °C, then were allowed to regain moisture for 24 h. The BP branch/leaf powder and mobile nylon bag samples were collected and crushed for moisture, crude protein (CP), crude fiber (CF), neutral detergent fiber (NDF), acidic detergent fiber (ADF), and total energy (GE) determination. Then, the nutrient apparent digestibility of the BP branch/leaf powder was calculated.

Dry matter (DM), % = 100% − moisture.

Digestible energy (DE), MJ/kg = GE − fecal energy.

Apparent digestibility of a certain nutrient, % = 100 × (intake of a certain nutrient − mass of a certain nutrient in feces)/intake of a certain nutrient.

#### 2.3.2. Growth Performance Measurement

During the first day of the experimental period, the donkeys fasted for 12 h and were then weighed the following morning to establish their initial body weight (IBW). Similarly, at the end of the trial, the donkeys fasted for 12 h and were weighed the next morning to determine their final body weight (FBW). The average daily gain (ADG) and the feed conversion rate for dry matter (FCR) were subsequently calculated.

#### 2.3.3. Collection and Measurement of Serum Samples

At the end of the trial, 10 mL of blood was collected from the jugular vein after fasting for 12 h. Serum samples were centrifuged at 3000 rpm for 10 min and stored at −20 °C for analysis.

Total protein (TP), albumin (ALB), globulin (GLB), triglyceride (TG), total cholesterol (TC), alanine aminotransferase (ALT), aspartate aminotransferase (AST), lactate dehydrogenase (LDH), γ-glutamyl transpeptidase (γ-GT), serum uric acid (UA), serum urea nitrogen (UREA), serum calcium (Ca), serum phosphorus (P), serum ammonia, immunoglobulin A (IgA), immunoglobulin G (IgG), and immunoglobulin M (IgM) were measured by Hitachi 7600 automated biochemistry analyzer (Hitachi Co., Tokyo, Japan). Serum superoxide dismutase (SOD), catalase (CAT), glutathione peroxidase (GSH-Px), total antioxidant capacity (T-AOC), and malondialdehyde (MDA) were detected using a commercial ELISA kit (Nanjing Jiancheng Bioengineering Institute, Nanjing, Jiangsu, China). And serum anti-tumor necrosis factor-α (TNF-α) was determined using a Multiskan MK_3_ microplate tester (Thermo Fisher Scientific, Waltham, MA, USA).

### 2.4. Statistical Analysis

Data processing and statistical analysis were performed using SPSS 20.0 statistical software. These data were tested for normality using the Kolmogorov–Smirnov test, then general linear model (GLM) analysis and Duncan’s multiple comparison were used to further analyze the data. All data were presented as mean ± standard deviation. Differences were considered significant at *p* < 0.05.

## 3. Results

### 3.1. Nutrient Level and Apparent Digestibility of Broussonetia Papyrifera Branch/Leaf Powder

The nutrient levels and apparent digestibility of the BP branch/leaf powder are presented in Table 2. The apparent digestibility percentages for dry matter (DM), crude protein (CP), crude fiber (CF), neutral detergent fiber (NDF), and acid detergent fiber (ADF) in BP branch/leaf powder are 51.88%, 67.27%, 64.86%, 49.59%, and 54.73%, respectively. The gross energy (GE) content of BP branch/leaf powder is 15.58 MJ/kg, with a digestive energy of 6.37 MJ/kg.

### 3.2. Growth Performance

As shown in Table 3, BP branch/leaf powder significantly affected the ADG of male Dezhou donkeys (*p* < 0.05). Donkeys in the 10% and 20% groups exhibited higher ADG and lower FCR. The ADG in the 20% group was significantly higher than that in both the control group and the 30% group (*p* < 0.05).

### 3.3. Serum Biochemical, Immune, and Antioxidant Indicators

#### 3.3.1. Serum Biochemical Indicators

As shown in Table 4, serum levels of AST, γ-GT, UA, UREA, Ca, P, and ammonia were not affected by dietary supplementation with BP branch/leaf powder (*p* > 0.05). The TP levels in the control group and the 30% BP powder group were significantly higher than those in the 10% group (*p* < 0.05). ALB levels in the control and 10% groups were significantly higher than in the 20% and 30% groups (*p* < 0.05). However, GLB levels in the 10% group were significantly lower than in the other groups (*p* < 0.05), and the A/G ratio was significantly higher in the 10% group compared to the others (*p* < 0.05). The 20% group showed lower TG levels (*p* < 0.05), and the control group had higher TC levels (*p* < 0.05), with no significant differences observed among the other groups (*p* > 0.05). Both the 20% and 30% BP branch/leaf powder diets significantly increased ALT levels, while LDH levels were lower in the 10% group (*p* < 0.05). No significant differences were observed in these parameters among the other groups (*p* > 0.05).

#### 3.3.2. Serum Immune Indicators

As shown in Table 5, donkeys in the 30% group had higher serum IgA levels (*p* < 0.05), while no significant differences were observed among the other groups (*p* > 0.05). The TNF-α level in the control group was significantly higher than those in the other groups (*p* < 0.05). Serum IgG and IgM levels were not affected by dietary supplementation with BP branch/leaf powder (*p* > 0.05).

#### 3.3.3. Serum Antioxidant Indicators

As shown in Table 6, serum SOD, CAT, GSH-Px, and MDA levels were not significantly affected by dietary BP branch/leaf powder supplementation (*p* > 0.05). T-AOC levels in the 30% group were significantly higher than those in the control group (*p* < 0.05), and there were no significant differences between the 10% and 20% groups (*p* > 0.05).

## 4. Discussion

### 4.1. Nutrient Level and Apparent Digestibility of Broussonetia Papyrifera Branch/Leaf Powder

In this study, the nutrient composition of BP branch/leaf powder was analyzed. The contents of CP, CF, NDF, ADF, and gross energy (GE) were found to be 18.68%, 45.45%, 41.75%, 41.06%, and 15.58 MJ/kg, respectively. Previous studies have reported that CP, CF, NDF, ADF, and GE values can range between 15.80 and 317.89%, 21.25 and 327.24%, 36.71 and 343.97%, 23.04 and 33.65%, and 17.94 and 18.76 MJ/kg, respectively [23]. The CP, CF, and ADF contents in the BP branch/leaf powder used in this experiment were higher than those reported in the study. Notably, the nutrient content varies across different parts of BP; the leaves have the highest CP content, while the branches have the lowest. In contrast, the CF, NDF, and ADF contents are highest in the branches and lowest in the leaves [24]. The significant differences between the BP branch/leaf powder used in our study and the one in previous research may be attributed to the varying proportions of BP branches and leaves.

The apparent digestibility of dry matter (DM), CP, CF, NDF, ADF, and GE, measured using the nylon bag method, were 51.88%, 67.27%, 64.86%, 49.59%, 54.73%, and 40.87%, respectively. An in vitro digestion test of hybrid BP indicated that the degradation rates of DM, NDF, and ADF could reach 48.08%, 72.26%, and 57.10%, respectively [25]. There is a significant difference from the results of this experiment, which may be related to the differences in animal species used and the nutrient content of BP.

### 4.2. Growth Performance

As an unconventional feed, BP boasts a high nutritional value, containing ample crude protein, fat, amino acids, and minerals. The crude protein content in its leaves can exceed 20% based on dry matter [17,26]. Studies have shown that the crude protein content of dried BP leaves is 6.7% higher than that of alfalfa and can even replace some soybean meal in diets [9,27,28]. In this study, the crude protein content of BP branch/leaf powder was 18.68%, compared to 19.78% in alfalfa. This lower value in BP branch/leaf powder, obtained from crushed branches and leaves, contrasts with higher values found in dried BP leaves in previous studies. The proportion of BP branch/leaf powder in the diet significantly influenced the average daily gain of Dezhou donkeys. When the proportion reached 20%, the average daily gain was significantly higher than that in the control group and the 30% group, possibly due to higher available nutrients. These results suggest that BP branch/leaf powder can partially replace alfalfa in the Dezhou donkey diet. Prior research on BP branch/leaf powder’s effects on donkeys is scarce.

Current research mainly examines the effects of BP silage on pigs, cattle, and other animals, with little focus on the direct crushing application of BP. Increasing BP silage in Holstein cow diets can significantly reduce dry matter intake and milk somatic cell count without markedly affecting milk yield, feed conversion rate, or milk protein content [29]. Adding 15% BP silage to beef cattle diets can significantly improve final weight, with average daily gain, dry matter intake, and feed conversion rate in the BP silage group surpassing those of the control group [21]. In this study, the suitable addition proportion of BP branch/leaf powder is higher than that of silage, possibly due to differences in properties. The BP branch/leaf powder is produced by crushing, maintaining the original properties of BP, whereas silage has a low pH that affects animal feeding and digestion. Consequently, the additional proportion of BP branch/leaf powder is slightly higher than that of silage. Additionally, the crude protein content in this experiment’s BP branch/leaf powder is lower than in alfalfa, so the substitution proportion should be moderate to avoid impeding growth. This experiment marks the first study on BP branch/leaf powder in donkeys or equine animals, holding significant potential for further research and promoting BP’s application in equine nutrition.

### 4.3. Serum Biochemical, Immune, and Antioxidant Indicators

Serum biochemical, immune, and antioxidant indicators are crucial for reflecting the health status of animals, significantly aiding in monitoring animal health and playing an important role in disease diagnosis and treatment. The serum levels of total protein, albumin, and globulin indicate dietary protein absorption and hydrolysis in the body, as well as the degree of the animal’s immune response [30,31]. Enzymes such as ALT, AST, LDH, and γ-GT provide insights into liver health and the extent of cellular damage, serving as vital indicators of liver function [32,33]. BP silage has been shown to increase the serum levels of total protein and globulin, decrease AST levels, and not significantly affect ALB levels [34]. In this study, the addition of BP branch/leaf powder influenced the levels of total protein, albumin, and globulin, but these returned to normal levels upon the addition of an appropriate amount without adversely affecting the body’s immune response. Notably, adding 20% BP branch/leaf powder significantly increased ALT levels, but did not significantly affect AST levels in serum, suggesting a specific impact on liver function that warrants further investigation. The serum levels of TG and TC are indicators of lipid metabolism and the body’s metabolic energy balance [35,36]. When BP branch/leaf powder was added at 20%, there was a significant reduction in the serum levels of TG and TC in Dezhou donkeys, possibly due to active substances in BP inhibiting certain lipid metabolism processes. BP silage can enhance the immunity of dairy cows by increasing serum IgA, IgG, IgM, and TNF-α levels [34]. In this study, the serum levels of IgA and TNF-α in Dezhou donkeys were significantly influenced by BP branch/leaf powder. A significant increase in IgA levels was observed in the 30% group, and a decrease in TNF-α levels was noted with the addition of BP branch/leaf powder, differing from previous results. This suggests that BP branch/leaf powder may have a specific effect on donkey immunity, potentially attributable to the unprocessed state of the BP branch/leaf powder, which could diminish the apparent effects of its immunity-enhancing active substances.

During cell metabolism, the body produces reactive oxygen species (ROS), which can damage biological macromolecules such as carbohydrates, proteins, lipids, and DNA. This leads to oxidative stress and a decline in physiological function, immunity, and production performance [37,38]. To counteract oxidative stress, the antioxidant defense system releases various enzymes, including CAT, GSH-Px, and SOD [39]. SOD acts as the first line of defense in the antioxidant system, catalyzing the conversion of the superoxide radical into hydrogen peroxide (H_2_O_2_). Subsequently, H_2_O_2_ is decomposed into water by CAT or GSH-Px [40]. MDA, the final product of lipid peroxidation caused by ROS, reflects the extent of lipid peroxidation and cellular damage in vivo. An increase in MDA content indicates exacerbated cell damage [41,42]. T-AOC, a comprehensive index, measures the body’s antioxidant capacity, reflecting the dynamic balance between oxidants and antioxidants and the state of free radical metabolism [43]. It may cause oxidative stress reactions in the body if the body’s antioxidant defense system is significantly affected and undergoes significant changes. Therefore, it is necessary to conduct antioxidant research in the development of a new type of feedstuff application. BP is rich in bioactive substances like phenolic aldehydes and flavonoids, which have antibacterial and antioxidant activities and are beneficial to animal health when used in feed [44,45]. Studies have shown that BP leaves can serve as a new source of natural antioxidants [46]. The stem bark of BP has anti-inflammatory activity and may be a potential therapeutic agent for inflammatory diseases [47]. It has been reported that BP leaf and its extracts can modify lipid metabolism in animals [48]. BP silage has been found to reduce MDA content in the serum and enhance lipid peroxidation resistance in Holstein cows [20]. Additionally, BP silage can increase SOD and T-AOC content and decrease MDA content in the serum, thereby improving the body’s antioxidant capacity [21]. In this study, the addition of BP branch/leaf powder did not significantly affect the serum levels of SOD and MDA, possibly because the flavonoid activity in the powder was diminished due to it not being ensiled. However, T-AOC levels were improved, particularly at a 30% addition level, which was significantly higher than that of the control group. This improvement is likely due to the antioxidant substances in BP enhancing the antioxidant capacity of Dezhou donkeys.

## 5. Conclusions

This study demonstrated that the BP branch/leaf powder was found to enhance the growth performance, serum indices, and T-AOC in Dezhou donkeys. Notably, BP branch/leaf powder can be incorporated into donkey diets, with an optimal supplementation ratio of 20%, effectively replacing two-thirds of the alfalfa content.

## Figures and Tables

**Table 1:** Ingredients and nutrition levels of diets (air dry basis, %).

Item	Control Group	10% Group	20% Group	30% Group
Corn	20.0	20.0	20.0	20.0
Cottonseed meal	2.0	2.0	2.0	2.0
Peanut meal	4.0	4.0	4.0	4.0
Wheat bran	5.0	5.0	5.0	5.0
Corn DDGS	10.0	10.0	10.0	10.0
Corn germ meal	5.0	5.0	5.0	5.0
Limestone	1.5	1.5	1.5	1.5
Premix ^1^	2.5	2.5	2.5	2.5
Rice straw	20.0	20.0	20.0	20.0
Alfalfa hay	30.0	20.0	10.0	0.0
*Broussonetia papyrifera* branch/leaf powder	0.0	10.0	20.0	30.0
Total	100.0	100.0	100.0	100.0
Nutrient levels ^2^				
DM (%)	87.62	87.42	87.21	87.01
GE (MJ/kg)	15.16	15.19	15.21	15.23
CP (%)	14.24	14.13	14.02	13.91
CF (%)	16.35	17.94	19.53	21.11
NDF (%)	41.14	39.71	38.29	36.86
ADF (%)	27.94	26.78	25.62	24.46

^1^ The premix provided the following per kg of diets: vitamin A 12,000 IU, vitamin D_3_ 1500 IU, vitamin E 40 mg, vitamin K_3_ 3 mg, vitamin B_1_ 2 mg, vitamin B_2_ 4 mg, vitamin B_6_ 4 mg, vitamin B_12_ 0.01 mg, pantothenic acid 20 mg, nicotinic acid 30 mg, folic acid 1.7 mg, biotin 0.3 mg, Cu 20 mg, Zn 80 mg, Fe 100 mg, Mn 80 mg, I 0.6 mg, Se 0.2 mg. ^2^ All values were measured values. Except for DM, the others were air-dried. DM, dry matter; GE, gross energy; CP, crude protein; CF, crude fiber; NDF, neutral detergent fiber; ADF, acid detergent fiber.

**Table 2:** Nutrient level and apparent digestibility of *Broussonetia papyrifera* branch/leaf powder.

Item	Nutrient Level (%)	Apparent Digestibility (%)
DM	90.05	51.88
CP	18.68	67.27
CF	45.45	64.86
NDF	41.75	49.59
ADF	41.06	54.73
GE (MJ/kg)	15.58	40.87
DE (MJ/kg)	6.37	-

DE was a calculated value, while the others were measured values. Except for DM, the others were air-dried. DM, dry matter; CP, crude protein; CF, crude fiber; NDF, neutral detergent fiber; ADF, acid detergent fiber; GE, gross energy; DE, digestive energy.

**Table 3:** Influence of dietary *Broussonetia papyrifera* branch/leaf powder supplementation on growth performance of donkeys.

Item	Control Group	10% Group	20% Group	30% Group	*p*-Value
IBW (kg)	196.20 ± 10.23	193.00 ± 8.28	193.60 ± 8.23	192.00 ± 8.51	0.891
EBW (kg)	209.20 ± 11.86	206.80 ± 9.63	209.40 ± 8.35	204.60 ± 9.34	0.851
ADG (g/d)	361.11 ± 51.97 ^b^	383.33 ± 63.34 ^ab^	438.89 ± 30.43 ^a^	350.00 ± 31.67 ^b^	0.036
FCR	12.33 ± 1.73	11.66 ± 2.00	9.97 ± 0.68	12.52 ± 1.17	0.059

IBW, initial body weight; EBW, end body weight; ADG, average daily gain; FCR, feed conversion rate. ^a,b^ Means within row with different superscripts differ significantly (*p* < 0.05).

**Table 4:** Influence of dietary *Broussonetia papyrifera* branch/leaf powder supplementation on serum biochemical indicators of donkeys.

Item	Control Group	10% Group	20% Group	30% Group	*p*-Value
TP (g/L)	71.16 ± 3.06 ^a^	66.54 ± 1.80 ^b^	69.44 ± 1.59 ^ab^	72.68 ± 4.77 ^a^	0.035
ALB (g/L)	34.36 ± 0.70 ^a^	34.42 ± 1.15 ^a^	31.66 ± 0.91 ^b^	32.54 ± 1.48 ^b^	0.002
GLB (g/L)	36.80 ± 3.23 ^a^	32.12 ± 2.41 ^b^	37.78 ± 1.35 ^a^	40.14 ± 3.45 ^a^	0.002
A/G	0.94 ± 0.09 ^b^	1.08 ± 0.11 ^a^	0.84 ± 0.04 ^bc^	0.81 ± 0.05 ^c^	<0.001
TG (mmol/L)	1.61 ± 0.08 ^a^	1.69 ± 0.14 ^a^	1.52 ± 0.06 ^b^	1.73 ± 0.04 ^a^	0.009
TC (mmol/L)	0.74 ± 0.34 ^a^	0.60 ± 0.14 ^ab^	0.41 ± 0.12 ^b^	0.39 ± 0.12 ^b^	0.047
ALT (U/L)	3.82 ± 0.57 ^b^	4.26 ± 0.77 ^b^	5.28 ± 0.61 ^a^	5.36 ± 0.66 ^a^	0.004
AST (U/L)	349.22 ± 53.06	298.58 ± 16.41	302.80 ± 30.11	284.42 ± 34.93	0.060
LDH (U/L)	439.88 ± 33.75 ^a^	365.50 ± 37.86 ^b^	463.34 ± 43.75 ^a^	446.08 ± 43.69 ^a^	0.007
γ-GT (U/L)	18.33 ± 2.95	20.20 ± 2.54	21.39 ± 0.57	20.83 ± 2.55	0.227
UA (μmol/L)	9.40 ± 2.88	5.80 ± 0.84	8.60 ± 3.05	8.40 ± 2.51	0.157
UREA (mmol/L)	8.82 ± 0.93	9.68 ± 0.94	9.40 ± 0.43	9.54 ± 0.71	0.357
Ca (mmol/L)	3.63 ± 0.12	3.44 ± 0.86	3.45 ± 0.64	3.69 ± 0.38	0.862
P (mmol/L)	2.65 ± 0.10	2.74 ± 0.07	2.65 ± 0.03	2.68 ± 0.06	0.162
Serum ammonia (μmol/L)	115.02 ± 13.73	145.24 ± 26.71	124.27 ± 19.34	108.85 ± 18.59	0.055

TP, total protein; ALB, albumin; GLB, globulin; A/G, the ratio of albumin and globulin; TG, triglyceride; TC, total cholesterol; ALT, alanine aminotransferase; AST, aspartate aminotransferase; LDH, lactate dehydrogenase; γ-GT, γ-glutamyl transpeptidase; UA, serum uric acid; UREA, serum urea nitrogen; Ca, serum calcium; P, serum phosphorus. ^a–c^ Means within row with different superscripts differ significantly (*p* < 0.05).

**Table 5:** Influence of dietary *Broussonetia papyrifera* branch/leaf powder supplementation on serum immune indicators of donkeys.

Item	Control Group	10% Group	20% Group	30% Group	*p*-Value
IgA (g/L)	0.93 ± 0.11 ^b^	0.94 ± 0.09 ^b^	0.91 ± 0.07 ^b^	1.16 ± 0.10 ^a^	0.002
IgG (g/L)	8.27 ± 0.67	8.77 ± 0.82	8.04 ± 0.63	8.72 ± 0.92	0.390
IgM (g/L)	0.87 ± 0.08	0.86 ± 0.06	0.80 ± 0.06	0.84 ± 0.04	0.340
TNF-α (pg/mL)	27.23 ± 3.73 ^a^	23.43 ± 2.57 ^b^	22.35 ± 3.14 ^b^	21.84 ± 2.14 ^b^	0.043

IgA, immunoglobulin A; IgG, immunoglobulin G; IgM, immunoglobulin M; TNF-α, serum anti-tumor necrosis factor-α. ^a,b^ Means within row with different superscripts differ significantly (*p* < 0.05).

**Table 6:** Influence of dietary *Broussonetia papyrifera* branch/leaf powder supplementation on serum antioxidant indicators of donkeys.

Item	Control Group	10% Group	20% Group	30% Group	*p*-Value
SOD (U/mL)	78.54 ± 11.28	70.63 ± 5.81	80.26 ± 1.71	76.27 ± 3.34	0.153
CAT (U/mL)	4.29 ± 0.52	4.83 ± 0.63	4.23 ± 0.34	4.71 ± 0.77	0.305
GSH-Px (U/mL)	110.75 ± 26.81	89.51 ± 9.46	88.13 ± 8.90	110.56 ± 16.95	0.080
T-AOC (mmol/L)	0.20 ± 0.03 ^b^	0.22 ± 0.02 ^ab^	0.23 ± 0.02 ^ab^	0.25 ± 0.02 ^a^	0.046
MDA (nmol/mL)	4.30 ± 0.94	4.18 ± 0.28	4.39 ± 0.35	4.13 ± 0.81	0.922

SOD, superoxide dismutase; CAT, catalase; GSH-Px, glutathione peroxidase; T-AOC, total antioxidant capacity; MDA, malondialdehyde. ^a,b^ Means within row with different superscripts differ significantly (*p* < 0.05).

## Data Availability

Data are contained within the article.
